# Prolonged Neutropenia and Yellowish Discoloration of the Skin, But Not the Sclera, Following Excessive Turmeric Raw Root Ingestion

**DOI:** 10.7759/cureus.14754

**Published:** 2021-04-29

**Authors:** Rashid Abdel-Razeq, Sereen Iweir, Tala Awabdeh, Fareed Barakat, Hikmat Abdel-Razeq

**Affiliations:** 1 Internal Medicine, Istishari Hospital, Amman, JOR; 2 Office of Scientific Affairs and Research, King Hussein Cancer Center, Amman, JOR; 3 Internal Medicine, King Hussein Cancer Center, Amman, JOR; 4 Pathology and Laboratory Medicine, King Hussein Cancer Center, Amman, JOR; 5 School of Medicine, University of Jordan, Amman, JOR

**Keywords:** curcumin, turmeric plant, breast cancer, neutropenia, jaundice

## Abstract

The medicinal use of curcumin has gained popularity in recent years especially so among cancer patients undergoing chemotherapy. In this report, we describe the case of a 51-year-old female breast cancer patient who self-medicated on large amounts of turmeric root infusions while receiving chemotherapy. The patient presented with yellowish discoloration of her skin, but normal-colored sclera. She also had severe neutropenia, which persisted despite halting chemotherapy. When her white blood cell counts returned to normal, only after stopping her turmeric regimen, we determined that her neutropenia is associated with turmeric consumption making this the first report to establish this link. This report demonstrates that, as an alternative form of medication, curcumin consumption should still be monitored in cancer patients. We provide the visible sign of yellowish skin discoloration as a visible aid for healthcare providers in detecting turmeric consumption as a risk factor to be considered in differential diagnoses of unexplained neutropenia.

## Introduction

Clinical trials are currently underway to determine the applicability of the reported anti-inflammatory and antioxidant properties of curcumin, a principal constituent of the turmeric plant, in treatment of certain cancers [1]. Due to its wide availability and minimal reported side effects [2], the use of curcumin as a form of alternative medicine has thus gained significant popularity in recent years, albeit in an often crude form and home-made extracts, relying on its perceived safety. Herein, we report for the first time the unusual severe neutropenia and sole discoloration of the hands as a result of unregulated turmeric use that was discovered in a breast cancer patient.

## Case presentation

A 51-year-old female patient was diagnosed with breast cancer in August 2016. On presentation, she had a breast lump, a biopsy of which confirmed a diagnosis of invasive ductal carcinoma, grade II. The tumor was positive for both estrogen (ER) and progesterone receptors (PR). Additionally, the patient’s human epidermal growth factor receptor 2 (HER2) was positive, with a +3 score by immunohistochemical (IHC) staining. Further evaluation showed multiple bilateral pulmonary and liver metastases along with a highly suspicious active bone lesion in the proximal end of the right humerus. She had a history of hypertension that was controlled on bisoprolol and hydrochlorothiazide. She was also known to have thalassemia trait and negative family history for malignancy. She had no symptoms related to her disease and her Eastern Cooperative Oncology Group (ECOG) performance status was zero. The patient’s breast exam was negative except for a 3-cm left breast mass with a retracted nipple.

**Figure 1:**
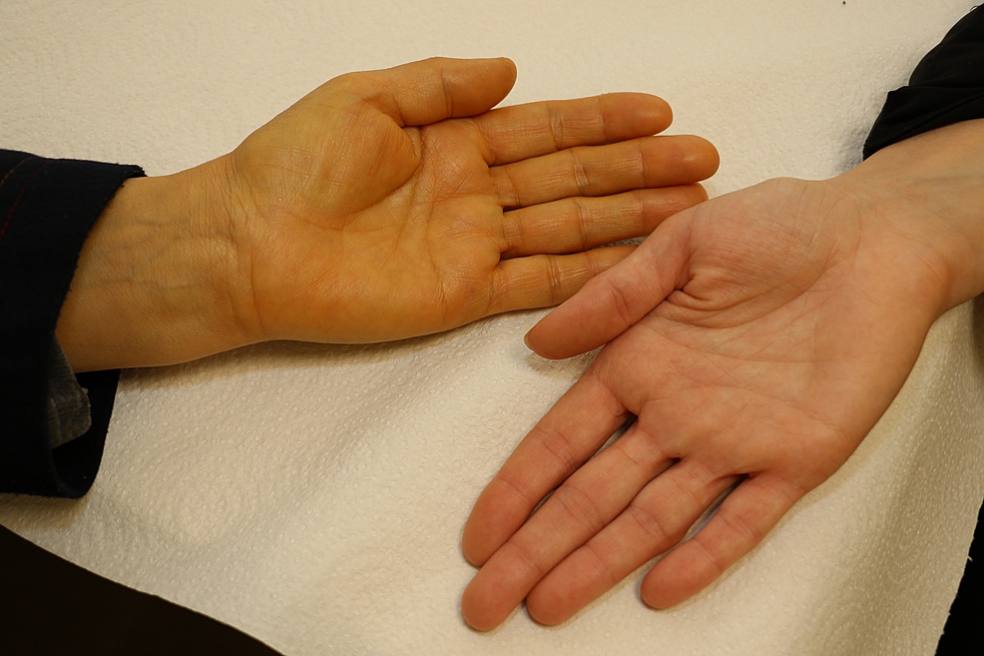
Yellowish discoloration of the skin. The patient's hand (left) showing yellow pigmentation in comparison to her daughter's hand (right).

Following detailed discussion with the patient, she was started on chemotherapy, initially with doxorubicin and cyclophosphamide, then her regimen was switched to docetaxel along with anti-HER2 therapy with both trastuzumab and pertuzumab, which resulted in almost total disappearance of both liver and lung lesions after the 6th cycle. Docetaxel was held after two more cycles, but both anti-HER2 drugs were continued along with endocrine therapy with an aromatase inhibitor. On routine clinic follow-up evaluation almost 6 months after the discontinuation of docetaxel, she was noticed to have a deep yellowish discoloration of her hands (Figure 1) but not the sclera, associated with no itching nor change in color of her urine or stool.

**Figure 2:**
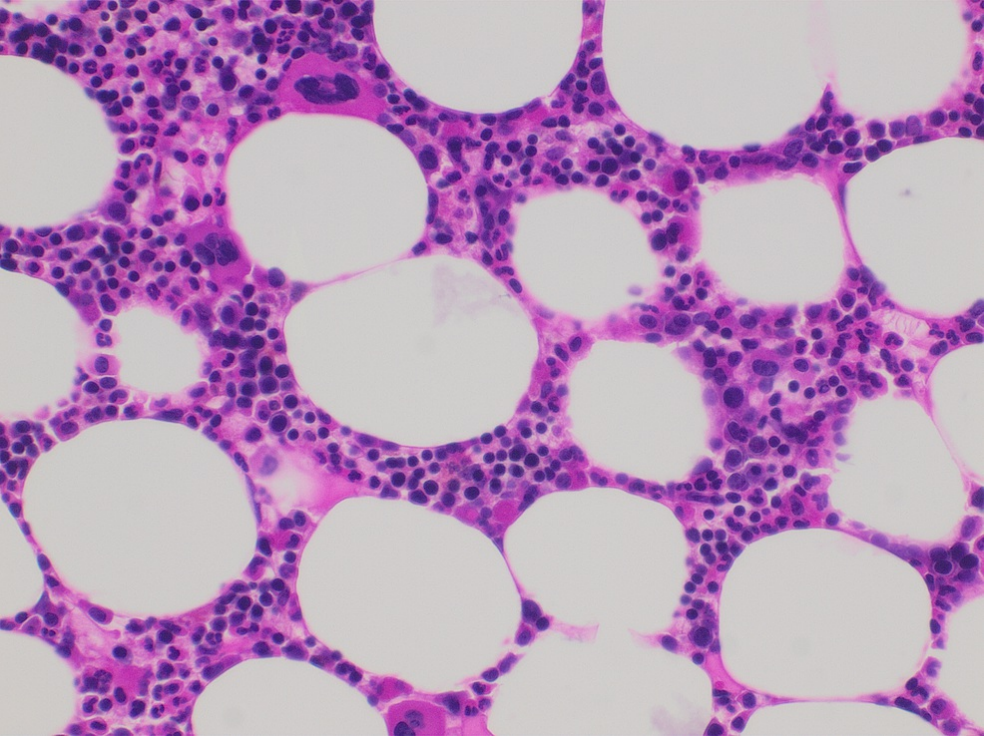
Bone marrow biopsy (H&E 4 x10). Erythroid predominance over granulocytes. The bone marrow is devoid of metastatic disease. The cellularity is rather normal except for granulocytic hypoplasia. Megakaryocytes are unremarkable. H&E:  hematoxylin and eosin.

**Figure 3:**
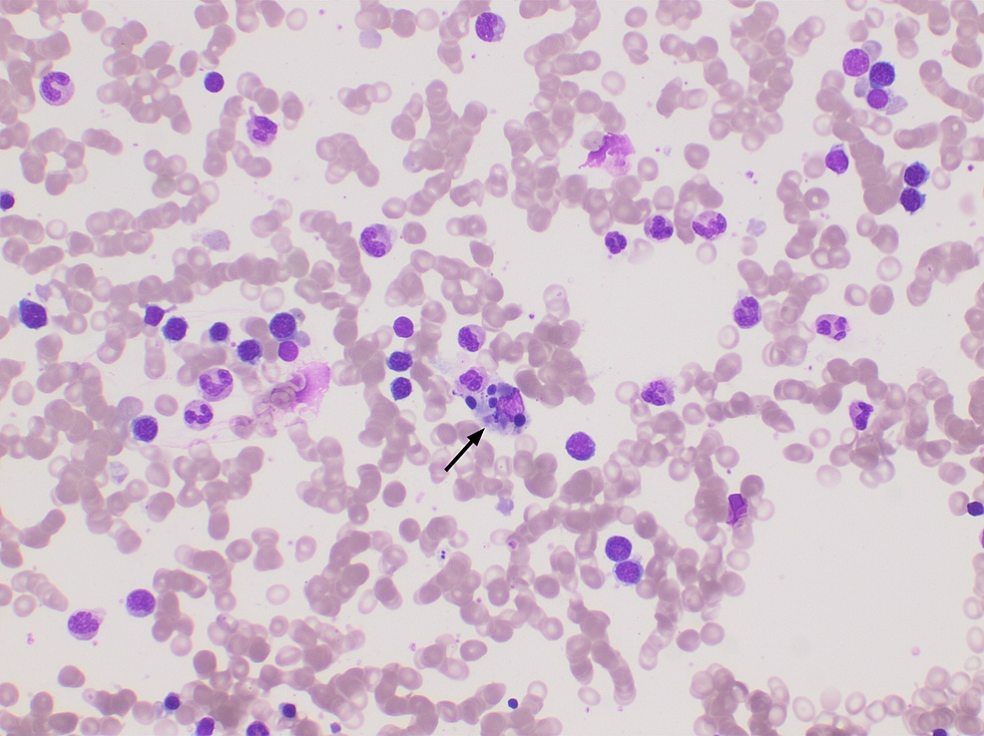
Bone marrow biopsy (Leishman stain 4 x10). Suppressed M:E (myeloid:erythroid) ratio with decreased granulocytic precursors. Note the presence of hemophagocytosis in the center of the field (arrow). The granulocytic and erythroid maturation is normal. Megakaryocytes are not depicted.

Imaging studies showed a significant increase in the size of the breast lump and mild increase in the liver lesions but without biliary dilatation. Her liver function tests showed a normal bilirubin, normal alkaline phosphatase (ALP), gamma-glutamyl transferase (GGT) and transaminase levels. Her kidney function was also normal. However, she had significant neutropenia with an absolute neutrophil count (ANC) that was <1000 cells/µL, because of which, chemotherapy was delayed. Her neutropenia persisted over a period of over eight weeks; therefore, a bone marrow biopsy was performed which showed normocellular bone marrow with granulocytic hypoplasia and no morphologic evidence of metastatic carcinoma or infiltrative marrow disease (Figures 2, 3).

**Figure 4:**
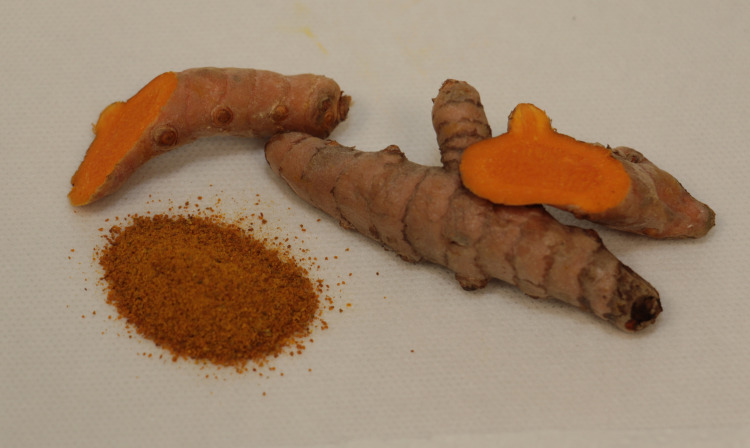
Turmeric root and powder. A photograph of the turmeric root and spice powder that were used by the patient.

On further questioning, the patient admitted that she had been consuming huge amounts of curcumin. She would drink 3-4 glasses of soaked fresh turmeric root and turmeric spice powder (Figure 4) on a daily basis since she stopped the docetaxel. The patient refused to stop her turmeric regimen and refused further chemotherapy. Three months later, with the persistence of her neutropenia, the patient agreed to stop consuming raw turmeric, and within four weeks, her total WBC and neutrophil counts returned to normal, while her skin discoloration persisted but with lower intensity. Chemotherapy was restarted and anti-HER2 therapy was continued with significant response and no major adverse events.

In accordance with the instructions of the institutional review board at our institute, the patient provided written informed consent to the publication of this case report and all of its accompanying images.

## Discussion

This case is yet another example that highlights the necessity for meticulous record-keeping by physicians and health care professionals (HCPs) of their patients’ use of complementary and alternative medication (CAM), especially among cancer patients. A common reality of CAM use is the fact that patients rarely disclose its details to their oncology treatment teams and other HCPs due to multiple reasons. Occasionally, health care team members fail to inquire about it, too [3]. Complementary and alternative medication use among cancer patients is gaining popularity, especially in developing countries such as Jordan; where 35.5% of cancer patients reported that they had used botanical CAM, the vast majority of which being in the form of crude herbal infusions (73.3%) as opposed to regulated dosage forms (6.8%) [4]. Furthermore, like 41.8% of Jordanian cancer patients, who base their decisions regarding CAM on the advice of their acquaintances [4], our patient had solely relied on the advice of her friends to self-medicate on turmeric and curcumin infusions.

Curcumin is a yellow pigment that is considered to be the active ingredient of turmeric, a flowering plant of the ginger family with the scientific name of *Curcuma longa* [5]. It is best known in its turmeric powder form as a spice used in curry (Figure 5) and is a polyphenol with proven anti-inflammatory properties [6]. Commercial curcumin supplements and extracts are available, and they contain 95% curcumin, while as a spice, turmeric powder is composed of only 3% curcumin [7]. Curcumin’s medicinal potential has captured the attention of the scientific community for years, due to its antioxidant, anti-inflammatory, and antiviral properties. For those reasons, curcumin has been extensively studied in in vitro and in vivo preclinical models for its potential use as an anti-cancerous agent [6], focusing on the anti-oxidative and modulatory interactions with cell-signaling pathways, such as its down-regulation of COX-2 and EGFR [8], and its inhibition of JAK2 activity [6,9]. A recent study, for example, demonstrated suppression of MCF7 breast cancer cell growth, via cyclin-D1 inhibition, upon treatment with curcumin nanoparticles [10].

On the other hand, clinical trials (CTs) investigating its efficacy in conjunction with traditional cancer treatment are relatively sparse and not as successful due to curcumin’s poor bioavailability [11]. Nonetheless, multiple CTs were commenced to determine the efficacy of curcumin addition to traditional treatment regimens, particularly for colorectal and pancreatic cancers [12,13]. In one recent randomized clinical trial, the efficacy and safety of curcumin was tested in patients with advanced breast cancer. In this study, 150 patients were randomized to receive either paclitaxel plus placebo or paclitaxel plus curcumin (300 mg intravenously weekly for 12 weeks). Compared to placebo, the overall response rate (ORR) was significantly higher in the curcumin group (51% vs. 33%, p < 0.01) [14]. Curcumin-induced side effects were reported to be restricted to gastrointestinal upset, while hematologic adverse events, like neutropenia, were not encountered. A recent review by Mansouri et al demonstrated that cancer patient survival rates were increased and their chemotherapy symptoms were reduced, depending on the dose of curcumin they received in curcumin-involving CTs of those patients [5]. Granted, previous studies on curcumin were conducted using regulated curcumin capsules, a study by Bayet-Robert, for example, prescribed a maximum dose of 8 g per day for seven days per each docetaxel treatment cycle, finding 6 g to be the maximal tolerable dose [15]. Our patient, however, was not only using an unregulated amount of turmeric root soaked in water on a daily basis, she revealed that she would also occasionally consume raw pieces of turmeric root.

Additionally, curcumin was reported to be an effective radio-sensitizing agent when administered with serum survivin to cervical cancer patients according to the results of a recent CT [16]. On the other hand, trials targeting breast cancer patients tend to focus on symptom alleviation rather than tumor reduction and reported improved quality of life measures and skin conditions when taking curcumin while receiving radiotherapeutic treatments [16]. An example being the CT conducted by Ryan et al, which reported reduced radiotherapy-induced dermatitis in breast cancer patients who received 6 gm curcumin capsules with their treatment [17].

Yellow pigmentation is a common occurrence in cancer patients, and is typically a sign of malignant intra-hepatic or post hepatic metastasis or injury. Jaundice is always accompanied by hyperbilirubinemia and usually occurs at a serum total bilirubin of approximately 3.0 mg/dL. Broadly, jaundice is caused by either hepatobiliary or hemolytic processes. The yellow pigmentation of the patient’s hands was noticed by her HCPs around the same time she was suffering from severe neutropenia. The fact that the patient’s sclera appeared normal; however, was nontypical of a patient with jaundice. Indeed, her normal ALP, GGT, and serum total bilirubin results, along with her normal stool and urine appearance, indicated that the patient in fact was not suffering from any liver injuries. In the literature, Xanthoderma is the term used to describe any yellow to yellow-orange discoloration of the skin, with jaundice and carotenoderma - a condition caused by consumption of carotenoids- being the primary causes [18]. The main factor differentiating carotenoderma from jaundice is the characteristic sparing of the conjunctivae in cases of carotenoderma, which is typically present in cases of jaundice if the bilirubin is at a level to cause skin findings [19]. Suspecting that the patient might have been consuming a carotenoid containing plant-based product, we began questioning her on her dietary routines, and if any she had made any adjustments to them since her diagnosis, initially reluctant, the patient divulged her turmeric consumption.

The lack of evidence of bone marrow infiltration (Figures 2, 3) led her team to rule out bone marrow metastasis during the same follow-up visit where her hand pigmentation was noticed. When the patient revealed that she had been consuming large amounts of turmeric roots, her physician suggested she stop her intake of curcumin, believing it to be the reason behind her yellowish skin to determine whether it is linked to her neutropenia, given their simultaneous manifestation. Unfortunately, the patient’s apparent lack of trust for modern medication and insistence on continuing with her turmeric regimen culminated in persistence of neutropenia and delay of appropriate therapy for three months. When the patient finally agreed to stop drinking soaked turmeric, her WBC returned to normal rapidly. Taken together, this sequence of events led us to conclude that her neutropenia was indeed caused by her unregulated curcumin intake. This was further confirmed by the fact that, so far, the patient has maintained usual neutrophil counts while resuming her scheduled chemotherapy cycles.

To our knowledge, this is the first report of curcumin’s effects on a cancer patient’s neutrophil count, since, to date, all previous data have restricted curcumin’s side effects to tolerable gastrointestinal symptoms. As for skin pigmentation, we found only one case report that associated yellowish coloration of a breast and thyroid cancer patient’s feet with oral intake of turmeric [20]. The visible sign of sole yellowish pigmentation of a patient’s limbs without accompanying jaundiced appearance of the patient’s eyes or face can serve as an indicator to oncologists and dermatologists of curcumin use, and if confirmed, HCPs can benefit by including that information in their differential diagnosis to determine the true cause of their patients’ neutropenia and avoiding unnecessary disruption of their treatment.

The popularity of the self-prescription of curcumin is aided by its significant accessibility, affordability, and presumption of safety. As a spice that is commonly used in Indian and Middle Eastern cuisines, our patient was quite confident with consuming it in the amount that she did. Nonetheless, given the ubiquity of both turmeric and curcumin supplements, and the multitude of articles touting their medicinal properties, the case of our patient will likely not be the last, and the visible signs of excessive curcumin consumption presented in this report may serve as a warning sign for HCPs in the future to ensure that they thoroughly question their patients on CAM use, and rule out turmeric as a cause for their patients’ neutropenia.

## Conclusions

Curcumin consumption, as a CAM, is commonly practiced especially among patients with cancer. Excessive use may lead to yellowish discoloration that may be confused with jaundice and may lead to prolonged neutropenia. A detailed history of CAM should always be sought.
